# Editorial: 3D Bioprinting of Vascularized Tissues for In Vitro and In Vivo Applications

**DOI:** 10.3389/fbioe.2021.754124

**Published:** 2021-09-06

**Authors:** Carmine Gentile, Khoon S. Lim, Giovanni Vozzi

**Affiliations:** ^1^ School of Biomedical Engineering/FEIT, University of Technology Sydney, Sydney, NSW, Australia; ^2^ Sydney Medical School, Faculty of Medicine and Health, University of Sydney, Darlington, NSW, Australia; ^3^ Light Activated Biomaterials (LAB) Group, Department of Orthopaedic Surgery and Musculoskeletal Medicine, University of Otago Christchurch, Christchurch, New Zealand; ^4^ Interdepartmental Research Center E. Piaggio Faculty of Engineering - University of Pisa, Pisa, Italy

**Keywords:** bioprinting, bioink formulation, vascularization, in vitro Models, disease models

3D bioprinting technology has fostered rapid and exciting advancements, such as the development of personalized *in vitro* disease models, high-throughput assays, and stem cell therapies for a range tissue engineering and regenerative medicine (TERM) application (Sun et al., 2020). It allows the precise dispense of cells and/or cell-laden biomaterials for constructing complex and functional living tissues or organs. Compared to traditional fabrication methods, 3D bioprinting offers an unprecedented ability to precisely control their material composition and cellular spatial distribution, producing tissues of high level of biomimicry in architecture and physico-chemical properties (Soliman et al., 2020; Sun et al., 2020). This Research Topic focuses on the usage of 3D bioprinting technologies to tackle one of the outstanding unmet challenges in the TERM field—vascularization, a critical factor for both the survival and function of cells within tissues/organs (Visconti et al., 2010; Klotz et al., 2019; Lim et al., 2019). The current major roadblock in clinical translation of lab-grown engineered tissues is the inability to incorporate functional vasculature within these constructs.

Chen et al. reviewed the current progress in using 3D-bioprinting technologies to fabricate vascularised tissues. The current limitations with 3D-bioprinting are the requirement to generate sufficient quantities of cells for a clinically and physiologically relevant construct, but also the need for construct maturation which can take up to few months prior to implantation. Although current research has showed that it is possible to embed tubular vessel constructs within *in vitro* tissue analogues, future attempts should focus on recreating the complex multi-scalar architecture of the vascular network to better mimic the inherent functionality. A functional vascular network supplies oxygen and nutrients to tissues through both macro and micro-circulation.

van Genderen et al. then showed the use of melt-electrowriting (MEW) to fabricate small-sized, porous and self-supportive tubular scaffolds. The scaffolds are flexible and able to be intertwined into multiple scaffolds, mimicking complex physiological situation such as renal tubes. The versatility of the manufacturing process allows control not only over the pore size but pore shapes as well. Cells seeded onto the scaffolds were able to bridge across the pores, form a dense basal luminal layer, which is then leak-free under perfusion. Overall, this study shows that the scaffold microstructure such as fiber spacing and pore shapes can influence cell functionality.

In terms of showing functionality of vascular networks formed *in vitro*, Nulty et al. showed the possibility of using *in vitro* pre-vascularisation approaches to enhance the *in vivo* mineralisation capacity of hypertrophic cartilage microtissues post implantation. By using a high-throughput microwell templating method, numerous hypertrophic cartilage microtissues can be fabricated, and then assembled into a vascular-promoting hydrogel to allow formation of a pre-vascular network. These pre-vascularised constructs were further implanted subcutaneously and found to promote more bone formation as compared to non-prevascularised constructs. With the recent advancements in 3D-bioassembly approaches (Mekhileri et al., 2018), these microtissues can be further coupled with biofabrication approaches, allowing 3D-bioprinting of patient-specific pre-vascularised implants.

Roche et al. explored how to bioengineer a vascular network within 3D bioprinted cardiac patches containing either human or mouse cells. In this study, the authors demonstrated that 4% alginate/8% gelatin (Alg/Gel) hydrogels are suitable to be used for the formulation of bioinks that contain single cells in suspension or within preformed 3D spheroids. Hydrogel printability and durability were compared using three extrusion bioprinters (two commercially available by CELLINK and Rokit Healthcare and one customised by REGEMAT3D). Printability was similar across the three platforms tested and patches were durable for at least 2 weeks in culture. Nevertheless, cells and spheroids were viable for at least 28 days in culture, presenting also contractile function when observed under a microscope. Most importantly, the endothelial cells network formed within 3D bioprinted patches was characterised by several level of complexity (from microcapillary to thicker vessels), which presented a hollow lumen as shown with the 3D rendering analysis by using IMARIS software. Altogether, these findings support the potential use of Alg/Gel hydrogels for the 3D bioprinting of viable and contractile cardiac patches for their transplantation *in vivo* to promote regeneration of the myocardium in cardiovascular disease patients.

The review by Hwang et al. discussed pathophysiological features typical of vascularized 3D bioprinted tissues. In this review article, first the authors highlight several approaches that focus on the generation of physiological *in vitro* models to mimic perfusion, different level of complexity in endothelization of blood vessel-like tissues, the volume-pressure relationship typical of *in vivo* blood vessels, and tissue-specific vascular networks achieved for renal and hepatic tissue engineering. The authors also describe the complex scenario of few examples of vascular disease state, following the treatment with drugs and cytokines known to induce toxicity and/or fibrosis and inflammation in the human body, or by changing other features in the 3D microenvironment, such as changes in oxygen and nutrient gradients or cell types used. Finally, the authors give an overview of how 3D bioprinting has been able to better mimic the tumor microenvironment heterogeneity, including its vascular network formation.

In summary, this Research Topic covers several aspects on how to promote vascularization using 3D bioprinting technology, with the aim to better mimic the tissue-specific microenvironment with improved cell survival and function. 3D bioprinting of vascularised tissues represents one of the latest advancements in the field to overcome hypoxia-driven cell death (necrosis and/or apoptosis) which is typical in the centre of any tissue thicker than 100–200 μm in diameter (Gentile, 2016; Roche et al.). In fact, there is evidence that the presence of a properly developed vascular endothelial cell network is able to prevent cell death in the centre of tissues even in absence of blood flow (Sun et al., 2020). Future studies focusing on the integration of 3D bioprinting technologies with patient-derived stem cells, microfluidics devices and tissue-specific biomaterials, will further allow engineering of functional vasculature to enhance drug discovery and personalized therapies for TERM purposes.

## References

[B1] Gentile C. (2016). “Filling the Gaps between the *In Vivo* and *In Vitro* Microenvironment: Engineering of Spheroids for Stem Cell Technology,” in Current Stem Cell Research & Therapy, 11, 652–665. 10.2174/1574888X10666151001114848 26423298

[B2] Klotz B. J. Oosterhoff L. A. Utomo L. Lim K. S. Vallmajo‐Martin Q. Clevers H. (2019). A Versatile Biosynthetic Hydrogel Platform for Engineering of Tissue Analogues. Adv. Healthc. Mater. 8, 1900979. 10.1002/adhm.201900979 PMC7116179 31402634

[B3] Lim K. S. Baptista M. Moon S. Woodfield T. B. F. Rnjak-Kovacina J. (2019). Microchannels in Development, Survival, and Vascularisation of Tissue Analogues for Regenerative Medicine. Trends Biotechnol. 37, 1189–1201. 10.1016/j.tibtech.2019.04.004 31076248

[B4] Mekhileri N. V. Lim K. S. Brown G. C. J. Mutreja I. Schon B. S. Hooper G. J. (2018). Automated 3D Bioassembly of Micro-tissues for Biofabrication of Hybrid Tissue Engineered Constructs. Biofabrication 10 (2), 024103, 2021. Available at: http://stacks.iop.org/1758-5090/10/i=2/a=024103. 10.1088/1758-5090/aa9ef1 29199637

[B5] Roche C. D. Brereton R. J. L. Ashton A. W. Jackson C. Gentile C. (2020). Current Challenges in Three-Dimensional Bioprinting Heart Tissues for Cardiac Surgery. Eur. J. Cardio-Thoracic Surg. 58 (3), 500–510. 10.1093/ejcts/ezaa093 PMC8456486 32391914

[B6] Soliman B. G. Lindberg G. C. J. Jungst T. Hooper G. J. Groll J. Woodfield T. B. F. (2020). Stepwise Control of Crosslinking in a One‐Pot System for Bioprinting of Low‐Density Bioinks. Adv. Healthc. Mater. 9 (15), 1901544. 10.1002/adhm.201901544 32323473

[B7] Sun W. Starly B. Daly A. C. Burdick J. A. Groll J. Skeldon G. (2020). The Bioprinting Roadmap. Biofabrication 12 (2), 022002. 10.1088/1758-5090/ab5158 32031083

[B8] Visconti R. P. Kasyanov V. Gentile C. Zhang J. Markwald R. R. Mironov V. (2010). Towards Organ Printing: Engineering an Intra-organ Branched Vascular Tree. Expert Opin. Biol. Ther. 10 (3), 409–420. 10.1517/14712590903563352 20132061 PMC4580374

